# Supplementary material for: Cytoplasmic Increase in Hsp70 Protein: A Potential New Biomarker of Early Infiltration of Cutaneous Squamous Cell Carcinoma Arising from Actinic Keratosis
Source: Cancers (Basel). 2020 May 3;12(5):1151. doi: 10.3390/cancers12051151 (PMC7281259; doi:10.3390/cancers12051151)

cancers

MDPI

Supplementary Materials:

Cytoplasmic Increase in Hsp70 Protein: A Potential New Biomarker of Early Infiltration of Cutaneous Squamous Cell Carcinoma Arising from Actinic Keratosis

Montserrat Fernández-Guarino, José Javier Zamorano León, Antonio José López Farré, Maria Luisa González Morales, Ana Isabel Sánchez Adrada, José Barrio Garde, Jose Antonio Arias Navalon **and Pedro Jaén Olasolo**

Table S1. Characteristics of the patients, controls and skin samples.

VARIABLE		SCC-AK GROUP (*n* = 10)	CONTROL GROUP (*n* = 10)
Gender (M/F)		6/4	6/4
Age		79.80 ± 1.41 *	47.9 ± 3.36 *
Phototype	I	1	1
	II	9	9
Solar exposition	Occasional	----	----
	Average	10	10
	High	----	----
Sample location	Axila-genitals	----	5
	Scalp-nape	-----	5
	Face-ear	5	-----
	Back hand	5	-------
AK overlying the SCC		10/10 (100%)	
AK at the edge of the SCC		9/10 (90%)	
Size (cm)		1,85 ± 0,11	
Dermis Infiltration (mm)		1,57 ± 0,35	
Level of infiltration (cm)		2,9 ± 0,94	
Foci of ulceration		1/10 (10%)	
Adnexal involvement		0/10 (0%)	
Elastosis in dermis		10/10(100%)	

Results are represented as mean ± SD. Abbreviations: M: male; F: female; SCC-AK: squamous cell carcinoma arising in AK; cm: centimeters; mm: millimeters; AK: actinic keratosis; SCC: Squamous cell carcinoma; * $p < 0.05$ with respect to SCC.

Table S2. Comparison of the protein expression profile in normal skin (controls) and SCC-AK.

PROTEIN		CONTROLS (*N* = 10)	SCC-AK (*N* = 10)	P VALUE
**Structural proteins**				
Actin		130.29 ± 49.14	271.60 ± 80.55	0.102
Annexin I		75.40 ± 37.00	44.72 ± 16.37	0.876
Annexin IV				
	Isoform 1	47.59 ± 14.51	53.20 ± 18.27	0.684
	Isoform 2	32.68 ± 19.96	15.63 ± 8.99	0.400
Annexin V				
	Isoform 1	39.95 ± 12.23	36.15± 8.51	0.905
	Isoform 2	35.65 ± 14.63	35.39 ± 8.96	1.000
Cytokeratin				
	Isoform 1	29.26 ± 9.77	34.10 ± 7.58	0.497
	Isoform 2	26.99 ± 11.04	13.47 ± 2.89	0.431
Calreticulin				
	Isoform 1	28.21 ± 7.44	23.16 ± 7.15	0.497
	Isoform 2	28.79 ± 13.78	25.84 ± 10.51	1.000
	Isoform 3	20.29 ± 10.00	19.56 ± 11.72	0.898
**Heat shock proteins**				
Hsp70		17.28 ± 4.11	42.20 ± 8.34	**0.035**
Hsp27		78.11 ± 49.03	308.50 ± 125.79	**0.006**
**Antioxidant protein**				
Glutathione-S-Transferase		24.67 ± 9.98	29.92 ± 6.48	0.195
**Tumor markers**				
Maspin		30.39 ± 12.15	22.86 ± 10.48	0.370
SCCA-2		83.78± 29.81	174.04 ± 50.00	0.423
**Transport proteins**				
Alpha-hemoglobin		32.63 ± 9.94	98.68 ± 18.11	**0.006**
Apo-AI		72.57 ± 23.43	74.79 ± 19.79	0.796
**Transcription factor**				
Rho-GDP		46.63 ± 19.63	27.53 ± 9.76	0.549

Results presented as mean ± SD. Abbreviations: SCC-AK: Squamous Cell Carcinoma over an AK; Hsp: Heat-shock protein; SCCA-2: Squamous Cell Carcinoma Antigen 2; Apo-AI: Apolipoprotein A1; Rho-GDP: Rho-Guanosine Triphosphate

Table S3. Spearman associations between level of SCC infiltration and the protein expression level of Hsp27, Hsp70 and alpha-hemoglobin.

PROTEINS	CORRELATION (Spearman. rho)	P VALUE	IHQ Hsp70 Nucleous	IHQ Hsp70 Citoplams
**All SCC-AK levels of infiltration (*n* = 10)**				
Alpha-hemoglobin	0.273	0.446		
Hsp27	−0.176	0.627	Mild+ (10/10)	Moderate++ (7/3)<br>High+++ (3/3)
Hsp70	−0.212	0.556		
**Levels of infiltration II y III * (*n* = 6)**				
Alpha-hemoglobin	0.200	0.704		
Hsp27	0.429	0.397	Mild+ (6/6)	Moderate++ (3/3)<br>High+++ (3/3)
Hsp70	**0.829**	**0.042**		
**Level of infiltration IV * (*n* = 4)**				
Alpha-hemoglobin	0.400	0.600		
Hsp27	−0.400	0.600	Mild+ (4/4)	Moderate+++ (4/4)
Hsp70	−0.600	0.400		

* Classification in Clark levels; SCC: Squamous cell carcinoma over an AK.

Western Blot figure: Representative Western blot experiments showing the expression of Hsp70 protein in cytoplasm from control and SCC-AK samples. β-actin was used as loading control. Bar graphs show the cytoplasmic expression Hsp70 levels of all the Western blots. Results are represented as mean±SD. *p<0.05 with respect to control.

Figure 2

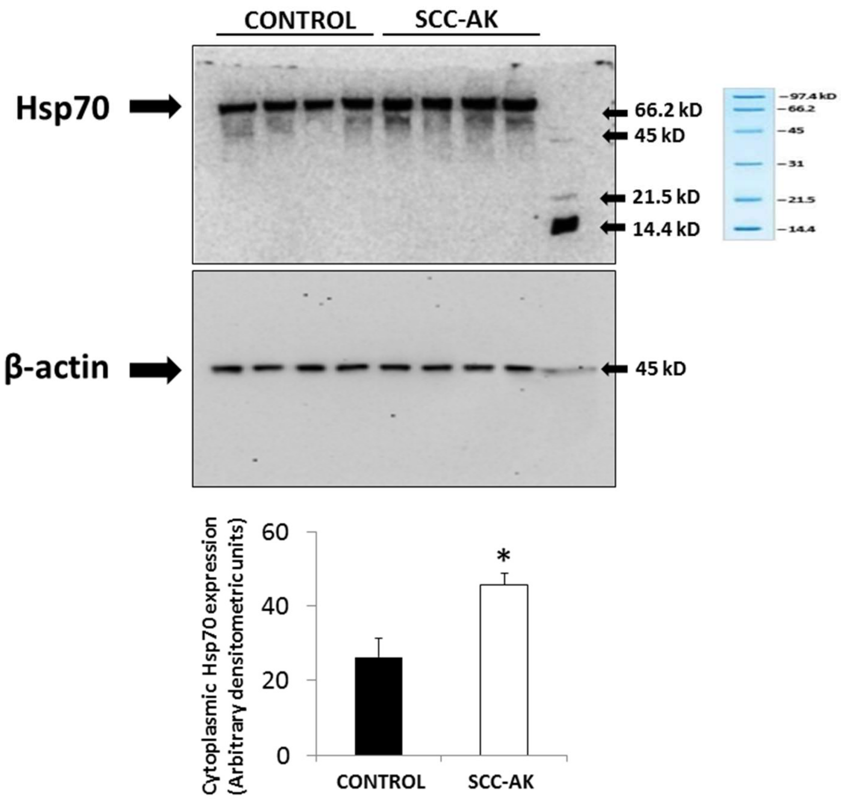

Supplement: Supplementary file 1 [file cancers-12-01151-s001.pdf]
